# Connectivity of Pathology: The Olfactory System as a Model for Network-Driven Mechanisms of Alzheimer’s Disease Pathogenesis

**DOI:** 10.3389/fnagi.2015.00234

**Published:** 2015-12-15

**Authors:** Katherine H. Franks, Meng Inn Chuah, Anna E. King, James C. Vickers

**Affiliations:** ^1^Faculty of Health, Wicking Dementia Research and Education Centre, University of Tasmania, Hobart, TAS, Australia

**Keywords:** Alzheimer’s disease, olfaction, denervation, enrichment, connectivity, amyloid beta

## Abstract

The pathogenesis of Alzheimer’s disease (AD) has been postulated to preferentially impact specific neural networks in the brain. The olfactory system is a well-defined network that has been implicated in early stages of the disease, marked by impairment in olfaction and the presence of pathological hallmarks of the disease, even before clinical presentation. Discovering the cellular mechanisms involved in the connectivity of pathology will provide insight into potential targets for treatment. We review evidence from animal studies on sensory alteration through denervation or enrichment, which supports the notion of using the olfactory system to investigate the implications of connectivity and activity in the spread of pathology in AD.

## Introduction

The olfactory system, used to detect odors in the environment, has been proposed to decrease in function due to physiological changes during normal aging (Doty, 2009; Masurkar and Devanand, 2014). Declines in olfactory function have also been commonly observed in neurodegenerative diseases, such as Parkinson’s disease (Hawkes et al., 1997; Doty, 2012), Huntington’s disease (Barresi et al., 2012), and frontotemporal dementia (Heyanka et al., 2014). Although some studies suggest that anosmia is associated with the presence of Lewy bodies rather than Alzheimer-type pathology, declines in olfactory function are commonly researched in Alzheimer’s disease (AD) (Mesholam et al., 1998). As well as decreased olfactory function, post mortem studies have demonstrated the presence of amyloid-beta (Aβ) and hyperphosphorylated tau (key products in the two major AD pathological hallmarks; Attems et al., 2014) in the olfactory system. Olfactory dysfunction can occur even at a preclinical stage of the disease (Masurkar and Devanand, 2014), suggesting that the olfactory system and its involvement in normal aging and AD may allow for a unique investigation of preclinical detection of AD. There is also some evidence to suggest that other risk factors of AD may interact with the olfactory network. For example, apolipoprotein E (apoE), a predictive risk factor for AD (Masurkar and Devanand, 2014), may be involved in olfactory function, as apoE knockout mice demonstrated poorer olfactory performance when compared to control mice (Nathan et al., 2004). Further, it has been shown that specific neural networks in the brain may be more vulnerable to preferential impacts of neurodegenerative diseases, such as AD (Seeley et al., 2009). Therefore, the olfactory system provides an ideal neural network to investigate the specific mechanisms that may be involved in olfactory dysfunction in normal aging and AD, as well as selective network-driven neuronal vulnerability in relation to AD-related pathology. This review aims to examine the involvement of the olfactory system in AD, and the possible role of the connectivity and activity of this system in the spread of pathology based on the findings from studies altering sensory input of the olfactory system.

## Human Olfactory Dysfunction in Alzheimer’s Disease

It has been postulated that AD is associated with olfactory dysfunction as the disease progresses. Early-stage AD has been shown to result in lower-level deficits in odor detection (threshold sensitivity), as well as higher-order deficits in odor quality perception, such as discrimination and identification (Li et al., 2010). In a recent review of behavioral testing in human beings, the majority of studies showed a correlation between cognitive status and baseline olfactory test scores (Masurkar and Devanand, 2014). Additionally, magnetic resonance imaging has shown deficits in odor identification ability that are correlated with smaller hippocampal volume in individuals with mild cognitive impairment (MCI) and early AD. However, in this study, hippocampal volume was not found to be associated with cognitive impairment test scores (Kjelvik et al., 2014). Despite this, there has been a strong predictive utility of olfactory tests for progression from normal aging to MCI to AD (Masurkar and Devanand, 2014). In support of this, it has also been shown that AD and amnestic MCI patients display significant deficits in olfactory identification tests when compared to healthy elderly people (Bahar-Fuchs et al., 2011). However, regardless of the diagnostic status of the participant, the large majority had no subjective complaints of olfactory decline. This suggests that although impairments in olfaction may occur in preclinical stages of AD, such as amnestic MCI (Yaffe et al., 2006), the use of olfactory decline as the sole predictor of progression to AD is limited (Bahar-Fuchs et al., 2011). Further, participants in experiments who may have preclinical AD-related changes, but who may not complain of olfactory impairment, could be erroneously classified as “controls” (Masurkar and Devanand, 2014). The situation is complicated by the proposition that in healthy adults, aging is considered the strongest correlate of olfactory decline (Doty, 2009). Thus, further studies on olfactory dysfunction in AD are required, particularly to gain a better understanding of the cellular mechanisms that operate to induce AD pathogenesis in the olfactory system and elsewhere in the brain.

## Neuropathogenesis in the Olfactory System in Alzheimer’s Disease

**Figure 1:**
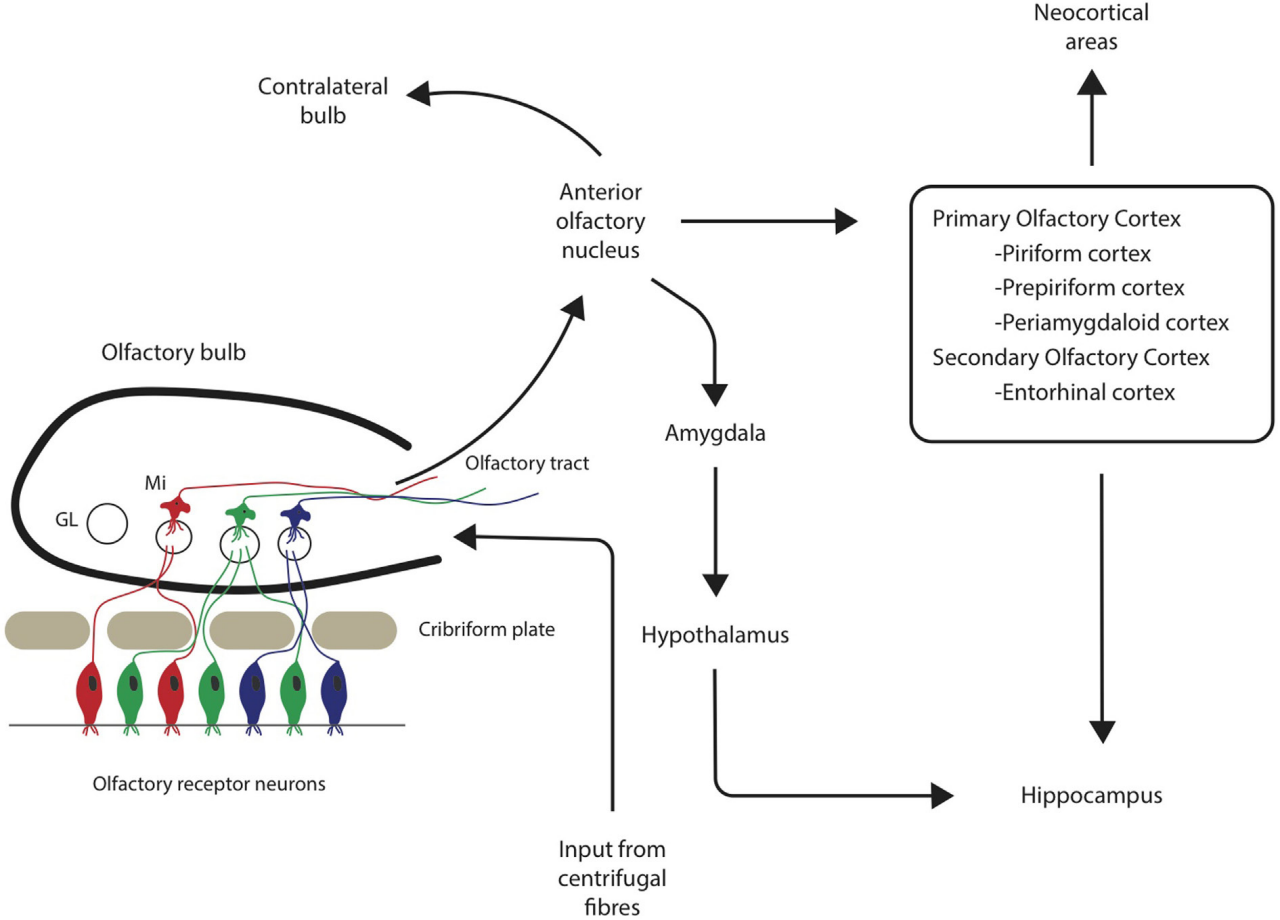
**Neuronal pathways from olfactory sensory neurons through the olfactory bulb and tract to associated cortical areas**. GL = glomerulus, Mi = mitral cells.

Both the olfactory system and areas of the brain with extensive connections to the olfactory system demonstrate pathological changes, such as Aβ plaques and neurofibrillary tangles (NFTs) (Christen-Zaech et al., 2003). The olfactory system is comprised of many different components, with the olfactory bulbs (OB) located at the base of the frontal lobe in human beings. Axons from the OB project to higher order, central structures in the primary olfactory cortex via the lateral olfactory tract (Doty, 2009; see Figure 1). Higher order structures include the anterior olfactory nucleus (AON), the piriform cortex (PCX), the anterior cortical nucleus of the amygdala, the periamygdaloid cortex, and the rostral entorhinal cortex. There are reciprocal relationships between these structures, as well as with other higher-order structures (Doty, 2009). For example, key efferents of the AON project to the ipsilateral OB and olfactory cortex, and also to the contralateral bulb and AON (Shipley et al., 2008). Beyond the primary olfactory cortex, neocortical areas, such as the lateral entorhinal cortex and the orbitofrontal cortex, receive substantial olfactory input (Wilson and Rennaker, 2010). Among mammalian species, the general circuitry of the olfactory–hippocampal pathway and its cytoarchitecture are preserved. There are outputs from the olfactory system that reach the parahippocampal region of the brain, including the perirhinal, parahippocampal, and entorhinal cortices (Eichenbaum, 1998). The primary olfactory cortex has connections to brain regions, such as the hippocampus (Haberly, 2001), which are also involved in AD. In later stages of the disease, NFTs are abundant in almost all components of the hippocampal formation, despite relatively few Aβ deposits (Braak and Braak, 1991). Specifically, hippocampal field CA1 is very severely affected by NFTs, whereas field CA3 and the subiculum are only somewhat affected (Price et al., 1991). Overall, the olfactory system has substantial connections to areas of the brain that are related to memory and display AD pathology.

In addition to hippocampal changes, AD pathological changes have also been described in olfactory cortical regions. For example, pathological changes that occur in the entorhinal cortex and associated neural systems may be responsible for early memory deficits in AD (Van Hoesen et al., 1991). Six clear stages related to the progression of Alzheimer-related changes have been determined based on the distribution of NFTs and neuropil threads in the brain, with the earliest changes involving the transentorhinal region of the cortex (Braak and Braak, 1991). Interestingly, studies have identified damage to the OB in the earliest stages of AD, even preceding damage to the entorhinal cortex (Kovács et al., 2001). These findings implicate the olfactory system in the early stages of disease progression, and also highlight the involvement of connectivity, both within the olfactory system and to associated areas, in AD pathology.

An early study into pathological changes in the olfactory system identified a higher number of NFTs and plaques in the olfactory cortex of human brains when compared to other brain regions. Further, these pathological markers of AD were also observed in other brain regions receiving input from the OB (Table 1; Reyes et al., 1993). Additional evidence also suggests that AD pathology and other related changes are present within higher cortical areas involved in olfaction. For example, computational brain imaging of MCI patients demonstrated that, over time, the patients who progressed to AD showed greater loss of gray matter in all brain areas than the MCI patients who remained stable, but the loss mainly occurred in the olfactory and polysynaptic hippocampal network (Prestia et al., 2010). Furthermore, in the AON, which is formed by large multipolar neurons embedded within the granule cell layer (GCL) and olfactory tract (Kovács, 2013), NFTs and a reduced neuron density have been identified (Esiri and Wilcock, 1984). NFTs have also been shown to be present in the AON and the parahippocampal gyrus in young (54–63 years of age), non-demented cases. However, with increasing severity of dementia, the number and density of tangles increased (Price et al., 1991). “Primitive” plaques were found particularly in the neocortex and, when dementia became more severe, there was a shift from “primitive” or diffuse plaques to “mature” plaques (Price et al., 1991). “Mature” plaques were described as having amyloid cores (Price et al., 1991), which have been classified as dense-cored plaques, in contrast to diffuse plaques (Dickson and Vickers, 2001). In addition, small numbers of plaques, as well as NFTs, have been found scattered throughout the AON in AD cases, but not in age-matched controls (Ohm and Braak, 1987). The research demonstrates that in human beings with AD, there will likely be a presence of NFTs in higher cortical areas, specifically the AON, and if the dementia is severe, also some plaques.

**Table 1:** **Pathological changes in various areas in AD cases**.

Diagnosis	Area	NFTs	Plaques	Neuropil threads	Age (years)	*N*	Reference
AD	Entorhinal cortex	Y	Y		61–89	10	Reyes et al. (1993)
	Prepiriform cortex	Y	Y				
	Periamygdaloid nucleus	Y	Y				
	Olfactory bulb and tract	Y	Y				
AD	Olfactory bulb	Y	X	Y	57–63	4	Ohm and Braak (1987)
	Anterior olfactory nucleus	Y	Y	Y			
Very mild/mildly demented	Anterior olfactory nucleus	Y	Y		78–95	6	Price et al. (1991)
	Entorhinal cortex	Y	Y				
More severely demented	Anterior olfactory nucleus	Y	Y		68–87	6	
	Entorhinal cortex	Y	Y				
AD	Anterior olfactory nucleus	Y	Y		61–85	10	Struble and Clark (1992)
	Olfactory bulb	Rare	X				
AD	Anterior olfactory nucleus	Y	NA		64–91	6	Esiri and Wilcock (1984)
	Olfactory bulb	X	NA				
Discrete AD	Olfactory bulb and tract	Y	X	Y	44–93	58	Christen-Zaech et al. (2003)
Moderate AD	Olfactory bulb and tract	Y	X	Y	53–90	14	
Severe AD	Olfactory bulb and tract	Y	Y	Y	44–93	19	
AD	Olfactory bulb	Y	Y (Aβ deposits)		74.8 (mean)	15	Kovács et al. (1999)
	Anterior olfactory nucleus	Y	Y (Aβ deposits)				

*Y = present, X = not present, NA = not reported/assessed*.

In the olfactory epithelium (OE) and OB, the pathological changes are less conclusive (Table 1). Some studies suggest that one of the earliest degenerative events in AD is the involvement of the OB and the olfactory tract (Christen-Zaech et al., 2003). However, others suggest that dysfunction in higher cortical areas can correlate with olfactory deficits in AD, either in addition to or without pathologic changes in the OB and the OE (Masurkar and Devanand, 2014). There are studies suggesting that in AD, NFTs are not present in the OB, specifically the glomerular layer (GL), mitral cell layer (MCL), or GCL (Esiri and Wilcock, 1984), and are rare in mitral and tufted cells of the OB (Struble and Clark, 1992). In contrast, other AD investigations have found NFTs in all layers of the OB except the outer fibrous layer (Ohm and Braak, 1987), accompanied by Aβ deposition and neuropil threads in all layers of the OB (Kovács et al., 1999). Further, NFTs have been found in the OB and olfactory tract of cases with severe, discrete, and moderate cortical changes, suggesting an early involvement of the olfactory system in AD (Christen-Zaech et al., 2003). Inconsistent findings have been reported in relation to plaques. Some studies failed to detect amyloid deposits (Struble and Clark, 1992) or plaques (Ohm and Braak, 1987) in the OB, while others have shown the presence of plaques in the OB and tract, mostly in cases with severe cortical involvement from AD (Christen-Zaech et al., 2003). Therefore, it appears that in human beings with AD, NFT burden in the OB is a more robust finding and is more consistent than the presence of amyloid plaques, particularly if the disease has not progressed to a severe level. This idea is supported by Braak staging, which suggests that plaque distribution can vary greatly between individuals, while NFTs have a more characteristic distribution pattern (Braak and Braak, 1991). Further, NFTs were present in almost all control cases between 67.5 and 81.1 years of age, suggesting that NFTs are an earlier pathological hallmark and that plaques may not present until AD progresses (Kovács et al., 1999).

One consideration is the recent *in vitro* and *in vivo* research suggesting that oligomeric Aβ may contribute to the neurotoxic events in early AD (Kirkitadze et al., 2002). For example, in rats, injections of conditioned medium containing soluble oligomeric Aβ transiently disrupted cognitive function (Cleary et al., 2005). In human AD cases, fibrillar oligomers are elevated and positively correlated with cognitive decline and neuropathological indices of amyloid plaque and tau tangle staging (Tomic et al., 2009). However, in the olfactory system, soluble fibrillar oligomers were not elevated in the OB of AD cases and were only higher in the OB of normal controls compared to other brain regions (Tomic et al., 2009). Aβ expression has been identified as being significantly more likely to be present in the OE of AD cases compared to normal cases or those with other neurodegenerative disease (Arnold et al., 2010). These levels correlated highly with averaged cortical Aβ plaque ratings; however, questions were raised as to whether the Aβ identified was fibrillar, as inclusions were similarly immunolabeled with an antibody against the N-terminus of Aβ, as well as an antibody labeling soluble oligomers (Arnold et al., 2010). Despite the evidence regarding oligomeric Aβ, fibrillar Aβ is likely to still play a role in AD (Cleary et al., 2005).

As detailed earlier, the olfactory system has well-defined connectivity both within the system and with other cortical regions. Research has previously suggested that neurodegenerative diseases, such as AD, target specific networks in the brain (Seeley et al., 2009). For example, the default mode hypothesis suggests that there is a relationship between default activity patterns in cortical regions and amyloid deposition in later life (Buckner et al., 2005). However, activity may not be the only determinant of network vulnerability. The connectivity within brain networks may partly explain the spread of neurodegeneration. Specifically, it has been proposed that there may be transneuronal transmission of pathology (Kapogiannis and Mattson, 2011). Based on the findings of studies investigating pathological changes in the olfactory system and connected areas, it is logical that the olfactory system is particularly suited to investigating mechanisms operating in a neuronal network that may drive neuronal vulnerability in AD. The olfactory system provides a novel approach to investigate the role of activity and connectivity on the spread of AD pathology, on a background of aging-related changes.

## Research into the Involvement of the Olfactory System in Mouse Models of Alzheimer’s Disease

Mouse models of AD allow the investigation of the effects of the disease and pathological changes in the olfactory system. Several mouse models of AD have been developed, including, for example, Tg2576, APP/PS1, and 5XFAD lines, with all models being based on the overexpression of a single or multiple amyloidogenic mutant human genes (Masurkar and Devanand, 2014). For example, Tg2576 mice overexpress a mutant form of human amyloid precursor protein (APP) containing a Swedish mutation (Hsiao et al., 1996), whereas APP/PS1 mice are double transgenic, expressing mutated forms of the APP and presenilin 1 (PS1) genes (Radde et al., 2006). However, there are some limitations to consider when using mouse models of AD and relating the findings back to human beings. For example, the temporal progression of the disease does not correlate well to AD in human beings (Masurkar and Devanand, 2014). Despite this, mouse models can still provide important insights into changes in the olfactory system in relation to AD-like pathology.

In the OE, olfactory receptor neurons (ORNs) have been shown to be impacted in mouse models of AD. For example, OMP-hAPP mice expressing APP in only mature ORNs or Gγ8-hAPP mice expressing APP in immature ORNs show apoptosis in the OE despite an absence of extracellular plaques (Cheng et al., 2011). Further, in 5XFAD mice, which are APP/PS1 mice that coexpress five familial AD mutations (Oakley et al., 2006), the olfactory nerve terminals are vulnerable to degeneration and this is associated with increased expression of amyloidogenic proteins prior to plaque appearance. This mouse model also demonstrated a decrease in glomerular area (Cai et al., 2012). Overall, studies propose ORN axonal dysfunction is involved in the activity-dependent pathophysiology of OB function by means of soluble Aβ (Masurkar and Devanand, 2014). Within the OB, non-fibrillar Aβ deposition has been observed in Tg2576 mice earlier than within any other brain area (Wesson et al., 2010a), supporting the proposition that the human OB is damaged very early in the disease progression (Kovács et al., 2001). Further, interneuron markers, such as somatostatin and calretinin, are also reduced in the OB and AON in APP/PSI mice (Saiz-Sanchez et al., 2013). This is consistent with the finding that, in APP mutant mice, increased Aβ production impairs neurogenesis in the subventricular zone (SVZ) (Haughey et al., 2002). Consequently, fewer interneurons, both periglomerular cells (PGCs) and granule cells (GCs), are generated via the rostral migratory stream (RMS) originating from the SVZ (Mobley et al., 2014).

Higher order cortical areas involved in olfaction also demonstrate pathological changes in AD mouse models. Specifically, the amount of Aβ deposition has been shown to be notably greater within principal olfactory areas, such as the OB and the PCX, when compared to the primary somatosensory and motor cortices (Wesson et al., 2010a). Other findings demonstrate that TAPP and Tg2576 mice display plaques in the olfactory cortex, cingulate gyrus, amygdala, entorhinal cortex, and hippocampus at only 6 months of age, becoming numerous in older mice (Lewis et al., 2001). TAPP mice were created by crossing Tg2576 mice with a line that develop tau-labeled inclusions (JNPL3), with tau-labeled inclusions being observed in the olfactory cortex, entorhinal cortex, and amygdala of TAPP mice as early as 6 months of age (Lewis et al., 2001). Similarly, 5XFAD mice first demonstrate plaques in deep layers of the cortex, whereas older mice develop amyloid deposits in the cortex, subiculum, and the hippocampus as well. Plaques were also present in the OB in the older mice, but to a lesser extent (Oakley et al., 2006), which is similar to that in human cases. From these findings, it is clear that in mouse models of AD, there are numerous pathophysiological changes in the olfactory system and connected areas.

## Insight into Mechanisms of Alzheimer’s Disease Pathogenesis Using the Olfactory Network as a Model

When considering findings from both human and animal studies of olfactory dysfunction in normal aging and AD, the results suggest that the olfactory system clearly displays physiological changes in both normal aging and in AD. In particular, in mouse models of AD, the olfactory system (specifically the OB) is vulnerable to early pathological hallmarks, such as the deposition of Aβ (Wesson et al., 2010a). According to the amyloid cascade hypothesis of AD pathogenesis (Hardy and Higgins, 1992), Aβ deposition in the brain triggers a series of events that ultimately leads to the development of the disease (Karran et al., 2011). For example, major downstream effects of Aβ deposition include synaptic and neuronal loss (Selkoe, 1991), as well as disruption to spontaneous activity of brain areas (Beker et al., 2014). This dysfunction is postulated to propagate to downstream neurons within the recurrent network (Beker et al., 2014), highlighting the idea that the olfactory system provides an ideal network for investigating mechanisms of AD pathogenesis.

Early symptoms of olfactory deficits in AD may stem from Aβ deposition in the OB, which then spreads progressively to connected cortical areas (Kovács et al., 2001; Wesson et al., 2010a). This may be particularly true if the connected areas are energetically challenged, such that these regions experience elevated excitation under oxidative stress or conditions of age-related impairment (Kapogiannis and Mattson, 2011). One hypothesis of the spread of AD pathology arose from the similarities to prion disease (Jucker and Walker, 2011). This is the view that AD pathology may be driven by progressive seeded aggregation of misfolded proteins, which spreads to interconnected neurons (Brettschneider et al., 2015). Support for a prion-like mechanism of transmission comes from findings that Aβ deposition can be induced by injection of brain extracts from human AD patients in mice that do not normally develop deposits (Morales et al., 2012). In this study, Aβ deposits were found in the cortex, far from the original injection site of the hippocampus. Thus, overall, connectivity may contribute to the spread of pathology in AD.

Deposition of Aβ in the OB is one of the earliest pathological changes in the olfactory system in the Tg2576 mouse model of AD (Wesson et al., 2010a). It has been shown that the level of olfactory behavioral dysfunction was strongly correlated with the amount of Aβ deposition in mouse models, specifically in a spatial and temporal manner (Wesson et al., 2010b). While Pittsburgh Compound B (PiB) PET scanning in human beings has shown that olfactory deficits in odor identification due to AD are not directly related to Aβ burden (Bahar-Fuchs et al., 2010), odor discrimination, and sensitivity were not investigated in this study. Further, it has been hypothesized that soluble Aβ oligomers may be the sole neurotoxic entity, although insoluble Aβ cannot be disregarded entirely (Haass and Selkoe, 2007). It has been demonstrated that soluble Aβ disrupts the OB network activity and olfactory function. For example, application of Aβ to OB slices of rodents resulted in decreased network activity of the OB at the GCL, in a concentration- and time-dependent manner (Alvarado-Martinez et al., 2013). Further, intrabulbar injections of Aβ resulted in decreases in olfactory function for at least 2 weeks, beginning 2 weeks after the application. It was also found that the OB is more sensitive to the effects of Aβ with increasing age (Alvarado-Martinez et al., 2013). Inversely, reducing the levels of soluble Aβ has been shown to restore olfactory cortical physiology and olfactory habituation ability (Wesson et al., 2011). Consequently, it is important to conduct further investigations to determine how Aβ may lead to olfactory dysfunction, as studies regarding the cellular mechanisms involved may provide insight into potential targets for therapy.

## Amyloid-β and Alteration of Synaptic Activity

It has been postulated that Aβ may play a crucial role in synaptic decline, specifically Aβ may be involved in controlling neuronal activity at specific synaptic types or neuronal networks (Palop and Mucke, 2010). This may be a reciprocal relationship, as neuronal activity has been shown to regulate the level of interstitial fluid (ISF) Aβ (Cirrito et al., 2005; Bero et al., 2011). This has led to the notion that Aβ is part of a feedback loop controlling neuronal excitability. For example, increased neuronal activity produces more Aβ, which in turn depresses synaptic function resulting in decreased neuronal activity (Kamenetz et al., 2003). High levels of Aβ would, therefore, act to suppress neuronal activity, while it has been suggested that intermediate levels of Aβ may enhance synaptic activity (Palop and Mucke, 2010). These findings may correspond to the observations that advanced stages of AD are linked to decreased neuronal activity (Swaab et al., 1998), while early stages or even prodromal stages of the disease are associated with hyperactivity (Zilberter et al., 2013).

In normal circumstances, Aβ is present in a soluble form in the ISF of the brain throughout life. These levels of extracellular Aβ are the precursor to Aβ aggregation and AD pathogenesis (Cirrito et al., 2005). Animal studies indicated that regional ISF levels in young mice are predictive of the regional amyloid plaque deposition in aged mice (Bero et al., 2011). There is also increasing evidence that Aβ levels can be modulated by synaptic activity. For example, ISF Aβ levels were shown to be directly influenced by synaptic activity, specifically synaptic vesicle release (Cirrito et al., 2005). This study postulated that it may be possible for physical and environmental changes to alter neuronal or synaptic activity in a region-dependent manner, thus modulating the level of Aβ that can accumulate in plaques.

Studies have been conducted to alter sensory input to determine what effect this may have on ISF Aβ levels. For example, using a Tg2576 mouse model of AD, unilateral vibrissae deprivation resulted in reduced ISF Aβ levels in the barrel cortex, whereas unilateral vibrissae stimulation increased ISF Aβ levels (Bero et al., 2011). Further, long-term unilateral vibrissae deprivation resulted in reduced levels of amyloid plaque formation and growth (Bero et al., 2011), suggesting that sensory input may modulate extracellular Aβ levels, impacting on the development of Aβ-related pathological hallmarks. In support of this, another study using an AD mouse model employed unilateral ablation of whiskers and chronic diazepam treatment to establish a chronic reduction of synaptic activity (Tampellini et al., 2010). Barrel cortices with decreased activity showed a decreased number of Aβ plaques and the area they covered. There was also an increase in Aβ_42_ immunoreactivity within neurons, but a decrease in the levels of synaptophysin, which may have reflected a decrease in the number of synapses (Tampellini et al., 2010). Surprisingly, despite a reduction in plaque load, spatial memory performance of mice undergoing the diazepam treatment continued to deteriorate. Therefore, while reducing sensory input may reduce Aβ pathology, it may not have any effect on behavior.

## Sensory Deprivation of Olfactory System

Many studies have experimentally manipulated synaptic activity of the olfactory system through various methods of deprivation, such as peripheral deafferantation, olfactory bulbectomy (OBX), and naris occlusion. The majority of these studies have focused on structural changes in the OB or neurogenesis (e.g., Couper Leo et al., 2000a,b; Ducray et al., 2002; Mandairon et al., 2003, 2006). In contrast, there have only been a few investigations of changes in AD-related pathology as a result of olfactory denervation (Table 2). Furthermore, these latter studies have produced some conflicting results. While studies on vibrissae deprivation and reduced activity in the barrel cortices indicate decreased ISF Aβ levels (Bero et al., 2011) and reduced plaque load (Tampellini et al., 2010), functional deprivation by unilateral naris occlusion in rats has been reported to result in an overproduction of Aβ in wild-type animals (Yan et al., 2007). This was postulated to be mediated by increased BACE protein levels, β-site cleavage activity, and Aβ_40_ levels in the deprived OB of wild-type rats, as there was no detected change in gamma-secretase (Yan et al., 2007). Similarly, functional deprivation in a transgenic mouse model of AD lead to a significant increase in BACE1 levels in the OB and PCX (Zhang et al., 2010). It is thought that this increase may facilitate amyloid plaque formation, supported by the increase in amyloid deposition, but not before the age that global plaque onset occurs in Tg2576 mice regardless of these levels (Zhang et al., 2010). These studies used naris cauterization to achieve sensory deprivation of the olfactory system. However, although this method results in structural changes, such as smaller ipsilateral OB size in adult rodents, it is argued that this method cannot achieve complete sensory deprivation [see Coppola (2012), for a review]. For example, studies demonstrating attenuated activity of specific cells in the OB after unilateral occlusion suggest that odors may pass into the contralateral nasal cavity (Philpot et al., 1997).

**Table 2:** **Changes in AD-related pathology due to olfactory denervation**.

Findings	Method of denervation		Species	Model	Age of treatment	Reference
Increased beta-amyloid levels in hippocampus and neocortex.	Bulbectomy	Bilateral	Mice	WT	6 months	Aleksandrov et al. (2004)
Increased tau phosphorylation in hippocampus.	Bulbectomy	Bilateral	Mice	Htau	3 months	Li et al. (2014)
Increased BACE1 levels and amyloid deposition in the OB and piriform cortex.	Naris occlusion	Unilateral	Mice	Tg2576	6 months	Zhang et al. (2010)
Increased BACE levels in deprived bulb.	Naris cauterization	Unilateral	Rats	Sprague-Dawley	?	Yan et al. (2007)
Decreased plaque load in ipsilateral OB and bilateral neocortex/hippocampus. Increase in APP positive cells in WT denervated OB.	Triton X-100	Unilateral	Mice	APP/PS1 WT	2–3 months	Bibari et al. (2013)

*WT = wild-type*.

An alternative method of olfactory denervation that results in greater sensory deprivation is OBX, which results in permanent loss of olfactory function (Mucignat-Caretta et al., 2006). Originally used in rodents as a model of depression (Kelly et al., 1997), it has also been shown to result in learning and memory impairments in rodents (e.g., Hozumi et al., 2003; Mucignat-Caretta et al., 2006; Yehuda and Rabinovitz, 2013). Thus, it was suggested that it is possible to produce an animal model of AD by using OBX, as the technique results in effects similar to AD symptoms (Yehuda and Rabinovitz, 2013). For example, mice that received a bilateral OBX at 6 months showed spatial memory deficits on the Morris water maze 6 weeks after the operation (Aleksandrov et al., 2004). Bilateral OBX in mice has also been shown to increase AD-related pathology. For example, as well as demonstrating spatial memory deficits, wild-type mice that received a bilateral OBX also showed increased levels of Aβ in the neocortex and hippocampus. This occurred around the same time as the onset of spatial memory deficits, reaching a level comparable to the concentrations seen in transgenic APP mice with early-stage plaque formation (Aleksandrov et al., 2004). However, while Aβ levels increased further in older transgenic mice, a similar level of Aβ was not reached in wild-type OBX mice (Aleksandrov et al., 2004). Another study employing bilateral OBX in a human tau mouse model showed that OBX increased both hyperphosphorylation and insolubility of tau in the hippocampus, suggesting that olfactory deprivation may hasten tau pathology (Li et al., 2014).

In contrast to the findings from the aforementioned studies proposing that olfactory deprivation using unilateral naris occlusion or bilateral OBX result in increased pathological changes, a recent study using a Triton X-100 solution to unilaterally denervate the OB found decreased AD-related pathology (Bibari et al., 2013). Triton X-100 is a method of OB denervation that has been used by many studies focused on structural changes following denervation rather than changes to AD-related pathology (e.g., Nadi et al., 1981; Verhaagen et al., 1990; Herbert et al., 2012). It has been shown that Triton X-100 selectively destroys mature ORNs and their projections, while leaving the remaining olfactory mucosa intact (Cummings et al., 2000). Therefore, it is a method of reversible peripheral deafferentation, in which the pattern of axon reinnervation from the OE to the OB begins to resemble control patterns after only 3 weeks (Cummings et al., 2000). APP/PS1 mice receiving repeated unilateral Triton X-100 nasal washes were found to have decreased plaque load in the ipsilateral OB and bilaterally in the neocortex and hippocampus (Bibari et al., 2013). Further, there was also an increase in the number of APP positive cells in the denervated bulb of wild-type mice, but not APP/PS1 mice, which could be due to pre-existing high levels of APP in the transgenic mice (Bibari et al., 2013). The findings of this study could be a consequence of downregulation in synaptic activity. Further research, particularly employing a bilateral method of denervation, is required to gain insight into the underlying mechanisms.

The differences in findings between the unilateral naris occlusion and bilateral OBX relative to the unilateral chemical denervation of the OB may be partly explained by the different methods of sensory deprivation. It has been argued that nasal occlusion leads to ORNs remaining viable, with increased immunoreactivity for olfactory marker protein (OMP) in the deprived bulb, labeling mature ORNs (Waguespack et al., 2005). Further, there are also increased levels of olfactory-specific adenylyl cyclase, which is activated by odorants as part of olfactory transduction (Coppola et al., 2006). Compared to Triton X-100, OBX is a large surgical insult that results in loss of olfactory inputs to other regions (Mucignat-Caretta et al., 2006). Findings from studies employing OBX may be due to the anterograde degeneration of neurons in brain regions that in normal conditions receive projections from the OB (Harkin et al., 2003). Further, OBX may also involve destruction of centrifugal innervation from several parts of the brain, including hippocampal structures and the olfactory cortex (De Olmos et al., 1978; Luskin and Price, 1983). The finding that OBX may increase β-amyloid levels in mice (Aleksandrov et al., 2004) may be due to damage to the brain. For example, in human beings, traumatic brain injury has been shown to accelerate Aβ deposition (Sivanandam and Thakur, 2012) and levels of Aβ_42_ in the CSF (Raby et al., 1998). As OBX removes the OB in its entirety, this method may not be ideal to investigate the role of connectivity within the olfactory system on AD pathogenesis, as the OB are a key component of this system. Additionally, findings of upregulation of BACE1 and accelerated amyloid deposition due to unilateral naris occlusion may be a result of compensatory upregulation of different molecular components from the olfactory transduction pathway, possibly as a result of increased activation of the non-occluded side of the OB (Bibari et al., 2013). Therefore, peripheral deafferentation using Triton X-100 may be a better approach to investigate AD-related changes in the olfactory network system.

## Olfactory Stimulation

While there are multiple studies investigating the effects of olfactory deprivation on AD-related pathology, there are limited studies investigating the effect of olfactory stimulation. While olfactory stimulation studies exist (e.g., Rochefort et al., 2002; Mandairon et al., 2011; Bonzano et al., 2014), only a few have a particular focus on AD-related pathology. Liao et al. (2012) showed that short-term olfactory enrichment (40 days) did not alter the levels of tau phosphorylation in rats, while long-term (80 days) olfactory enrichment attenuated the phosphorylation of tau in areas of the hippocampus and cortex; the effect of odor enrichment on the OB was not examined (Liao et al., 2012). Due to the shortage of studies primarily focusing on the effect of olfactory stimulation on AD-related pathology in the olfactory system, future studies should investigate these effects employing the odor enrichment protocols from previous studies.

It is relevant to investigate the effects of olfactory stimulation or enrichment because it can be regarded as an aspect of environmental enrichment. It has been argued that environmental enrichment leads to neural changes, for example, increase in synaptogenesis and cell survival in neurogenesis (van Praag et al., 2000). Further, passive exposure to odors results in behavioral changes, such as improving olfactory discrimination (Mandairon et al., 2006), suggesting that physiological changes induced by enrichment can be translated into behavioral changes. Consequently, such neural changes may also modulate Aβ amyloidgenesis. While some studies have demonstrated that environmental enrichment may lead to increased amyloid burden in transgenic mice (Jankowsky et al., 2003), it may also build resistance to the negative effects of Aβ (Jankowsky et al., 2005). Further, studies using transgenic mice have shown that environmental enrichment can lead to decreases in the levels of steady state cerebral Aβ peptides and amyloid deposition (Lazarov et al., 2005). Therefore, there may be a connection between synaptic activity, APP processing, and Aβ production, which is modulated by environmental enrichment. Conversely, it has also been hypothesized that increased synaptic activity from enrichment may increase susceptibility to Aβ deposition if there are high levels of APP present (Cirrito et al., 2005). This is an important hypothesis to test using the olfactory system, as previously, it has been demonstrated using electrophysiological techniques that in an AD mouse model, the OB experiences hyperactivity in early life (Wesson et al., 2011). This hyperactivity corresponds to Aβ deposition restricted to the OB and may increase PCX activity, thus leading to impaired olfactory perception. Later in life, PCX dysfunction corresponds to hypoactivity, perhaps due to upregulation of Aβ production in both the OB and PCX (Wesson et al., 2011). These findings highlight that connectivity, as well as activity, of the olfactory system may play some role in Aβ deposition. It has also been suggested that Aβ can lead to GABAergic dysfunction, which may contribute to aberrant synchrony in neural networks, translating to disruption of cognitive functions (Palop and Mucke, 2010). The organization of newly generated inhibitory interneurons and their connections may be directed by OB activity, as their survival is upregulated by odor exposure (Rochefort et al., 2002). Thus, the effect of environmental enrichment, such as odorant stimulation on AD pathology, is a complex issue that is poorly understood and warrants further investigation.

## Olfactory Dysfunction and Alzheimer’s Disease Pathology

While the causal relationship between Aβ and olfactory dysfunction remains somewhat unclear, it may be an interactive relationship, rather than a one-way effect. For example, increased Aβ may drive olfactory dysfunction, but olfactory dysfunction may also result in increased Aβ production. Further, it is worthwhile to consider the interactive effects of NFTs, which may also influence olfactory dysfunction in AD. Not only have decreases in olfactory function been shown in mice overexpressing tau (Macknin et al., 2004), but also tau pathology has been reported in the olfactory system of individuals with AD. For example, studies have identified tau pathology in the OB and olfactory tract (Attems and Jellinger, 2006), and the AON (Esiri and Wilcock, 1984; Tsuboi et al., 2003), even in early stages of the disease (Tsuboi et al., 2003; Attems and Jellinger, 2006). The olfactory system presents an ideal model to address these issues, specifically aiding in the investigation of the network-driven mechanisms of AD pathogenesis, potentially through altering sensory input in mouse models of AD.

## Sensory Input and Neurogenesis

The olfactory system is unique, as even in adulthood, related neural regions have the ability to regenerate. While not demonstrated in human studies, transgenic mouse models of AD have shown that cell proliferation and neurogenesis is impaired, particularly in the SVZ (Rodriguez and Verkhratsky, 2011). Neurogenesis occurs in two regions of the olfactory system – peripherally in the OE (Schwob, 2002) and centrally in the OB (Haughey et al., 2002). Even into adulthood, the OE contains a population of proliferating progenitor cells (Schwob, 2002). ORNs continually grow, die, and regenerate in a cycle lasting 30–60 days (Sultan-Styne et al., 2009). Mature ORNs degenerate due to nerve injury or from exposure to environmental agents that enter the nasal cavity. The degenerated neurons are replenished by continuous neurogenesis among the basal cells of the OE (Huard and Schwob, 1995). Not only do ORNs regenerate in adult animals, there is also continual generation of new interneurons (PGCs and GCs) in the adult brain (Mobley et al., 2014), which are predominately GABAergic inhibitory interneurons (Parrish-Aungst et al., 2007). As well as peripheral neurogenesis in the olfactory system, neurogenesis occurs centrally in the OB, which receives newly generated neurons from the SVZ. The OB is connected to the SVZ by a large migratory pathway known as the RMS (Lledo et al., 2008). While the RMS was originally difficult to define in human beings, recent findings suggest that the adult human RMS is actually similar to other species, including the rat (Kam et al., 2009). The SVZ contains stem cells that have the ability to proliferate and differentiate into neurons in the OB (Haughey et al., 2002). Specifically, cells migrate to the GCL or GL to differentiate into interneurons (Luskin, 1998).

There is increasing evidence to suggest that alterations to sensory input can modulate neurogenesis in the adult OB. Specifically, sensory deprivation by axotomy of mature ORNs to produce unilateral reversible deprivation in wild-type mice, revealed an increase in both cell death and proliferation in the SVZ, RMS, and OB within the first 2 weeks of denervation, returning to levels of controls after 1 month (Mandairon et al., 2003). However, it has been suggested that it is not merely olfactory exposure that alters neurogenesis; rather olfactory learning is responsible (Alonso et al., 2006). This has been supported by findings that demonstrate learning of an odor discrimination task increased the number of newly generated cells in the deep, but not superficial, GCL. Further, there was a critical period between 18 and 30 days when new cells were more likely to survive, corresponding to the time when the new cells are starting to form glutamatergic synapses (Mouret et al., 2008). Therefore, it may be that olfactory learning helps newly generated inhibitory interneurons in the OB to survive by altering synaptic connections. This is important to consider, as disruptions to the balance between the excitatory and inhibitory neurotransmitters in the brain (glutamate and GABA) are present in AD (Kapogiannis and Mattson, 2011). While studies such as these provide important models to investigate the effects of altering sensory input in the OB, it is also important to consider that not all cellular mechanisms are dependent on sensory input.

## Summary

Overall, current evidence suggests that, in AD, specific networks in the brain may be more vulnerable to the pathology of the disease. They may provide a model for network-driven mechanisms of AD pathogenesis. The olfactory system is one such network that has been implicated in the early stages of AD. Not only have pathological hallmarks been identified in this system, but they are also prevalent in connected cortical regions, well known for their involvement in AD. While connectivity may help to explain the development or progression of the disease, alterations to neuronal activity have also been suggested as being involved, particularly in relation to amyloid deposition. Although studies have begun investigating the impact of sensory alteration through denervation or enrichment, there has not been a heavy focus on AD-related changes, thus the mechanisms behind the disease pathogenesis remain elusive. This review has discussed the implications of connectivity and activity in the spread of pathology in AD and proposes that the olfactory system is a well-defined network that can be used to address these issues in future studies.

## Author Contributions

All authors had substantial input into the conception and design of the review. KF was the main writer of the review and produced the tables and figures. JV, MC, and AK had roles in critiquing and improving drafts of the manuscript produced by the first.

## Conflict of Interest Statement

The authors declare that the research was conducted in the absence of any commercial or financial relationships that could be construed as a potential conflict of interest.
